# Surgical consent, perception of the patients who underwent a surgical operation in the Kurdistan region, Iraq

**DOI:** 10.1186/s12910-025-01218-0

**Published:** 2025-05-02

**Authors:** Dawan Jamal Hawezy

**Affiliations:** Department of Surgery, Faculty of General Medicine, Koya University, Kurdistan Region KOY45 Koya, Iraq

**Keywords:** Informed consent, Patient, Surgery, Surgeon, Anesthesia

## Abstract

**Introduction:**

Patient satisfaction is a significant measure of healthcare service quality as the patient is the center of any surgical procedure. Patient satisfaction refers to the extent to which a patient’s expectations of optimal care align with their perception of the care received. Patient satisfaction during informed consent is enhanced when written informed consent is accompanied by verbal consent in the preoperative period. Satisfied patients are more inclined to adhere to therapy, engage actively in their care, utilize healthcare services, willingly partake in decision-making, and remain with a healthcare provider. This research examines the practical and ethical considerations of obtaining informed consent during surgical procedures. To better understand and make informed decisions, this study aims to assess the efficacy of present consent methods and pinpoint obstacles patients encounter.

**Methodology:**

A cross-sectional study was conducted from April to December 2024. Data were gathered by second-year students from Koya University’s Faculty of General Medicine by interviewing postoperative patients who had undergone general surgical procedures. The results were entered into a Google form and analyzed using SPSS27.

**Results:**

In interviews with participants, 430 out of 572 patients (75%) indicated trust in their surgeons performing the surgery, while 525 patients (91%) expressed respect for their surgeons’ opinions. However, 41% (239 patients) reported not reading the informed consent form, and a similar percentage denied that the details of the form were explained by the medical staff responsible for the surgery as there are some medical terms or situations in the form that are challenging to assume if not explained.

**Conclusion:**

Compared to others, participants with a higher educational level sought extensive time from the responsible surgeons to discuss every detail of the surgery before signing the informed consent, with a statistically significant difference observed. A similar difference was noted between private and public hospitals.

**Supplementary Information:**

The online version contains supplementary material available at 10.1186/s12910-025-01218-0.

## Introduction

Surgical interventions are essential in addressing various medical conditions, encompassing routine and complex cases. Despite advancements in surgical procedures, the potential for postoperative complications remains a substantial concern, adversely affecting the overall positive trajectory of surgery [1]. The ramifications of surgical problems can be extensive, endangering a limb, an organ, or even the patient’s life; hence, surgical decisions carry significant ethical implications. Indeed, a characteristic aspect of surgery is the injury placed on the patient from the initial incision. Ideally, the surgery’s benefits outweigh the surgical wound’s detriments; however, the ethical maxim typically attributed to Hippocrates is not significantly applicable to surgeons, who inevitably inflict pain as part of their vocation. Many surgeons regard the assumption of personal responsibility for one’s acts and omissions as an essential component of surgical training [2].

As the patient is the center of any surgical procedure, Patient satisfaction is a significant measure of healthcare service quality. Patient satisfaction refers to the extent to which a patient’s expectations of optimal care align with their perception of the care received. Patient satisfaction during informed consent is enhanced when written informed consent is accompanied by verbal consent in the preoperative period. Satisfied patients are more inclined to adhere to therapy, engage actively in their care, utilize healthcare services, willingly partake in decision-making, and remain with a healthcare provider [3].

An optimal setting for acquiring informed consent for surgery is a private and confidential atmosphere that allows the patient to interact openly with the healthcare professional without interruptions. This allows the patient to inquire, voice concerns, and obtain comprehensive explanations regarding the surgical procedure. Informed consent should be obtained well in advance of the scheduled surgery. Patients should be afforded adequate time to examine the facts offered, confer with family or trusted individuals, get a second opinion if needed, and arrive at an informed decision without experiencing haste or pressure [4]; acquiring informed consent from patients prior to surgery constitutes the actual implementation of an interactive physician-patient interaction and deference to patients’ autonomy. The informed consent process is not merely a document to be signed; it is a procedure that requires respect for patients by offering comprehensive information to enable their voluntary decision-making concerning the proposed treatment methods [5].

Biomedical ethics pertains to the moral dimensions of surgery, which is crucial for fostering trust between physician and patient; surgeons have an ethical duty to guarantee that all information on the patient’s conditions and any suggested procedures is conveyed, ensuring the patient’s safety is not compromised. Biomedical ethics is an ever-evolving discipline, necessitating that physicians be abreast of advancements pertinent to their medical specialty. The cornerstone of medical ethics is founded upon the following fundamental principles: respect for persons, beneficence and justice [6].

The patient-surgeon relationship is fundamental for effective communication skills and is a needed aspect of sound medical ethics. Informed consent is essential for effective communication skills, ultimately improving patient satisfaction. This research aims to examine the practical, legal, and ethical considerations of obtaining informed consent during surgical procedures. To better understand and make informed decisions, this study aims to assess the efficacy of present consent methods, pinpoint obstacles encountered by patients, and provide solutions. In addition to addressing issues including educational variation and public-private sector differences, the research intends to evaluate the effect of informed consent on patient autonomy and satisfaction.

## Methodology

The Institutional Review Board approved the study conducted by the faculty of general medicine at Koya University, numbered 25/1006, on February 11, 2024, endorsing the research and the questionnaire. This questionnaire was specifically developed for this study, incorporating some concepts from previous research [6], and is included as a supplementary file.

A cross-sectional study was performed from April 2024 to December 2024 across six hospitals (four public and two private) in five cities within the Kurdistan region of Iraq; data were gathered by second-year students of the academic year 2024/2025 from the Faculty of General Medicine at Koya University, after validating and performing the pilot study. After surgery, they interviewed patients in their home language, explaining each question and recording their answers onto pre-made Google forms. For details of the questions and the way of collection, a training course was held for the students, and the researcher was in direct continuous contact with them.

The interview was conducted in Kurdish, while the Google form was completed in English. This form comprises two sections totaling 41 questions; the first section contains 16 questions addressing the patient’s demographic differences, including health status and surgical details, while the remaining questions pertain to the relationship between the patient and the operating surgeon, encompassing all aspects of perioperative care that the patient should understand before signing the informed consent.

Patients who underwent elective and emergency surgery were enrolled. Exclusions were made for surgeries outside the general surgery field and for patients under 18 years of age; additionally, patients hospitalized in the intensive care unit postoperatively, psychologically impaired patients, and patients who cannot speak or hear were also excluded.

### Data collection

A total of 572 patients who underwent different surgical procedures in the general surgery specialty were interviewed during their postoperative period, most of them on the same day of operation (day 0) and others on the second day.

Students enter the patient’s responses into the Google form, and data is analyzed using SPSS 27, with frequencies and chi-square tests.

## Results

Of the 572 patients interviewed, 339 were male, and 338 were between 18 and 40. Table 1 demonstrates the participants’ variability.

**Table 1:** Demographic information of the participants

topics	variables	number	percentage
sex	Male	339	59.3%
	Female	233	40.7%
age	18–40 years	338	59%
	40–60	126	22%
	60 and more	108	18.8%
Marital state	Married	351	61.15%
	Unmarried	206	36.1%
	Divorced and widow	15	2.61%
Educational state	Illiterate	151	26.3%
	Basic educational level	290	50.6%
	Higher educational level	131	22.9%
occupation	Academic staff	9	1.5%
	Employer	146	25.5%
	Housewife	25	4.3%
	Retired	51	8.9%
	Student	118	20.5%
	Worker	140	24.4%
	None	83	14.5%

Surgical procedures differ in different aspects, such as whether the operation is done through laparoscopy or the open method, the type of anesthesia used, and the type of hospital. Table 2 shows these differences: 464 patients operated by the open method, and only 15 underwent spinal anesthesia.

**Table 2:** Regarding patient/surgery detail

Variable		number	Percentage
Type of surgery	Open	464	81%
	laparoscopy	108	18.8%
Type of anesthesia	General	421	73.6%
	Local	136	23.7%
	Spinal	15	2.6%
Hospital	Private	188	32.8%
	Public	384	67.1%
Past medical history	None	419	73.2%
	Cardiovascular disease	87	15.2%
	Diabetes mellitus	17	3%
	Neurological disease	7	1.2%
	Cardiovascular and diabetes mellitus	30	5.2%
	Respiratory disease	12	2.1%

**Fig. 1:**
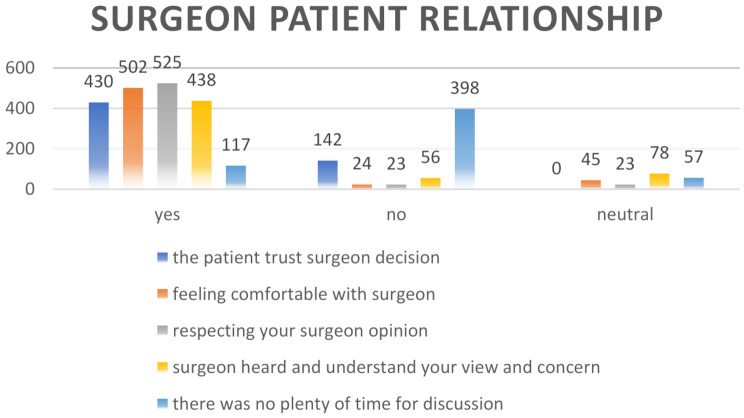
Surgeon-patient relationship

A crucial element of medical ethics, particularly in surgery, is the relationship between the patient and surgeon. After training, students conduct interviews with participants, exploring various facets of this relationship in depth and subsequently gathering their responses. Figure 1 demonstrates the different patterns of this relationship: 430 patients out of 572 trust the surgeons who performed their operation, while 525 respect their surgeons’ opinions.

**Fig. 2:**
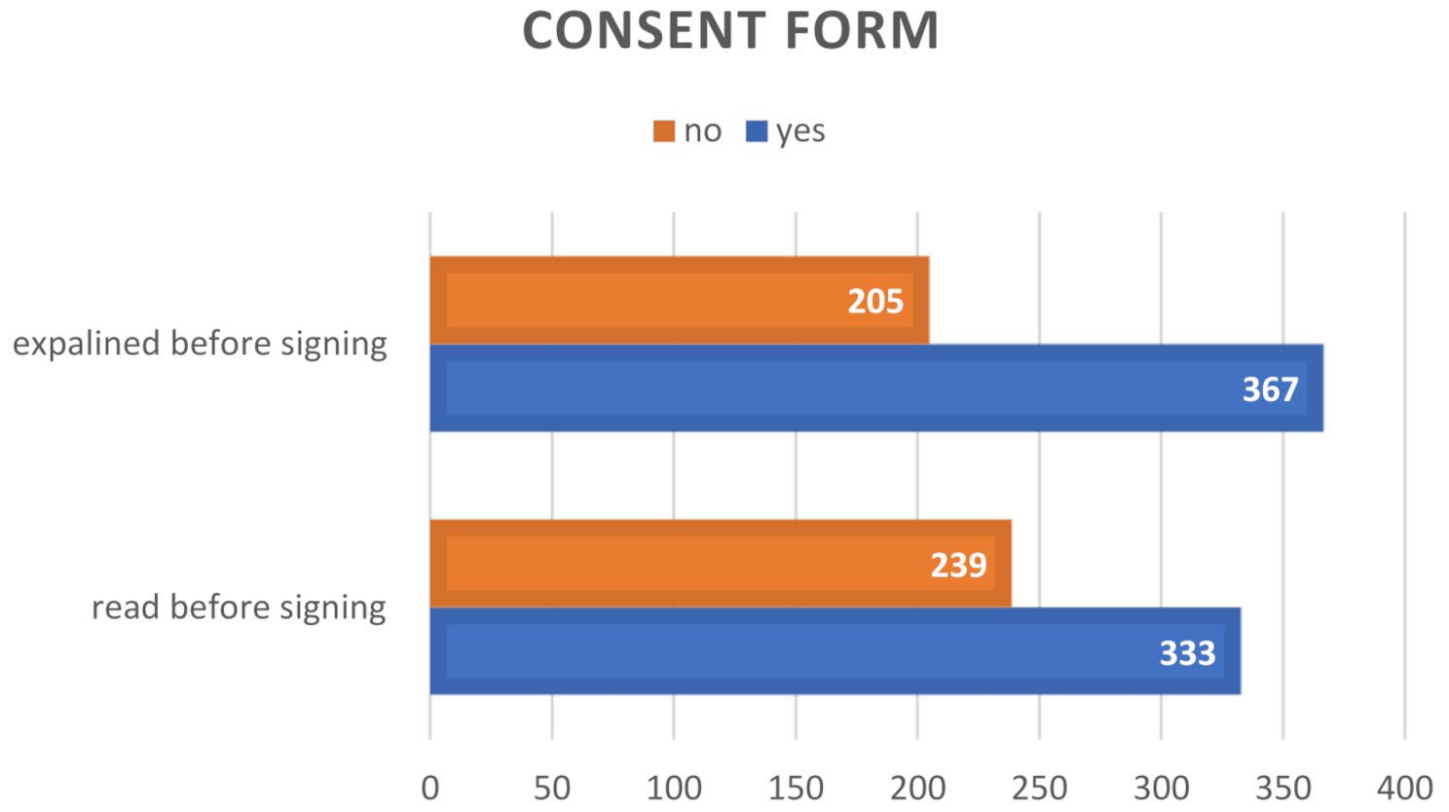
Explanation and reading of the consent form before signing

It is rational to review any document and seek expert clarification prior to signing; however, two hundred thirty-nine patients reported that they had not read the informed consent form, with a similar proportion asserting that it had not been adequately explained to them. Figure 2 illustrates specific details of that.

**Fig. 3:**
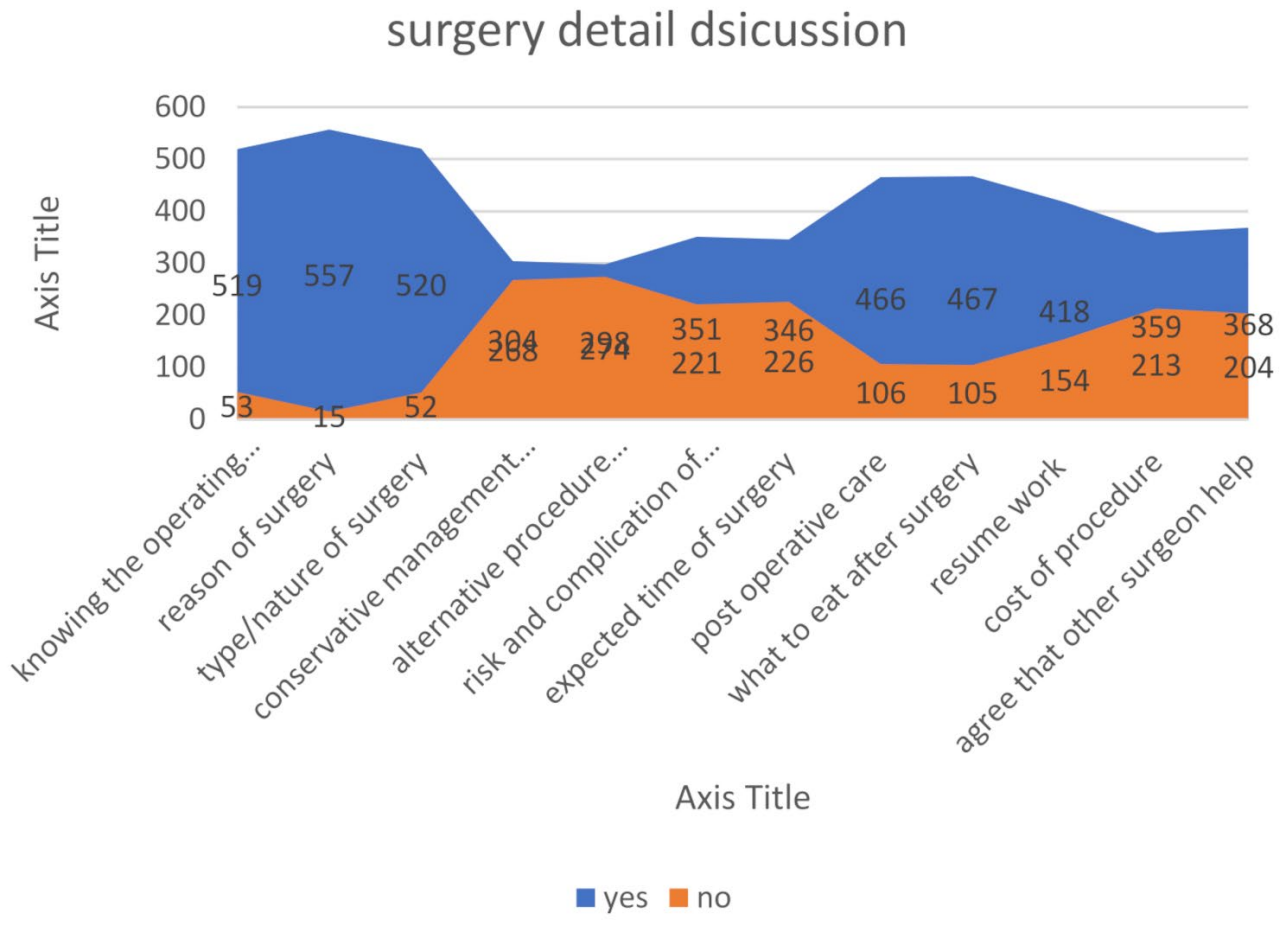
Surgery details discussion

The informed consent form in our locality explicitly states that the surgeon may seek assistance from another surgeon during the surgical procedure if necessary. However, despite this being stipulated in the consent form, a considerable proportion of patients refute the involvement of additional surgeons in their procedures. Refer to Fig. 3 for further details.

**Fig. 4:**
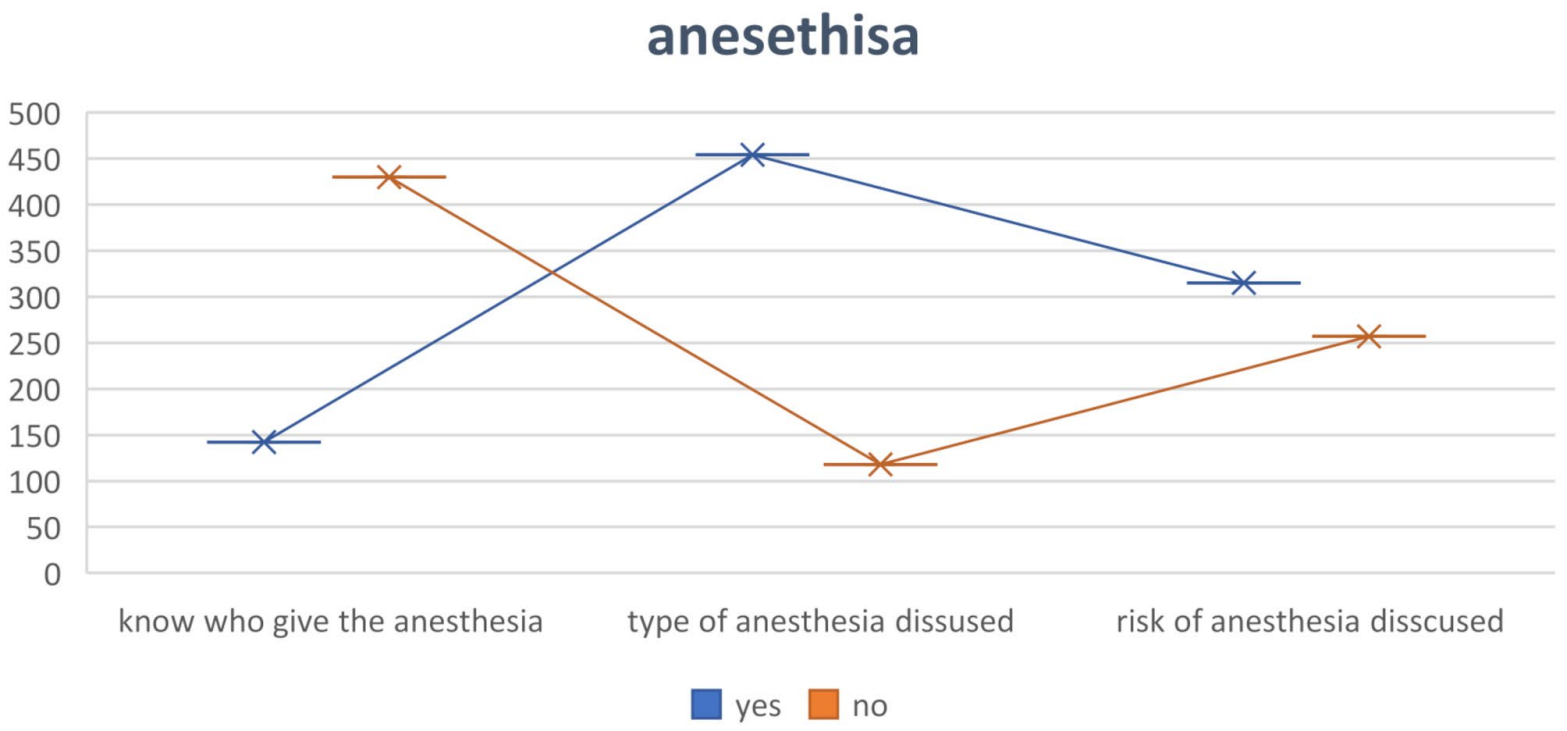
Participant knowledge about anesthesia

The use of anesthesia is crucial to all surgical procedures, and it is an ethical obligation for patients to be informed about the specifics of anesthesia, including the responsible professional, the type of anesthesia administered, and associated risks. Figure 4 demonstrates the responses of participants concerning these details.

The responsibility for discussing and obtaining surgical informed consent among healthcare workers in our locality remains unclear. During interviews, most participants indicated that the surgeon is responsible for this discussion. Table 3 presents these variables.

**Table 3:** Healthcare worker who explains the informed consent process

variable	frequency	percentage
Nurse	159	27.8
Senior house officer	44	7.7
Junior house officer	32	5.6
The surgeon performs the procedure	337	58.9
Total	572	100

We analyze the outcomes according to educational level, and Table 4 presents these details by calculating the *P*-value differences. There was a statistically significant difference among academic levels regarding allocated time for discussion.

**Table 4:** Different aspects of consent discussion according to educational level

	Educational level	No (%)	Yes (%)	Neutral(%)	Total (%)	*P* value
The patient trusts the surgeon’s decision	Illiterate	44 (7.69%)	107 (18.7%)	0	151 (26.3%)	0.336
	Basic education	69 (12%)	221 (38.6%)	0	290 (50.6%)	
	Higher education	29 (5%)	1 (0.1%)	0	131 (22.9%)	
	Total	142 (24.8%)	43 (7.5%)	0	572 (100%)	
Feeling comfortable with the surgeon	Illiterate	4 (0,6%)	137 (23.9%)	10 (1.7%)	151 (26.3%)	0.420
	Basic education	11 (1.9%)	255 (44.5%)	24 (4.1%)	290 (50.6%)	
	Higher education	9 (1.5%)	111 (19.4%)	11 (1.9%)	131 (22.9%)	
	Total	24 (4.1%)	503 (87.9%)	45 (7.8%)	572 (100%)	
Respecting the surgeon’s opinion	Illiterate	6 (1%)	140 (24.47%)	5 (0.87%)	151 (26.3%)	0.585
	Basic education	**11 (1.9%)**	263 (45.9%)	16 (2.79%)	290 (50.6%)	
	Higher education	6 (1%)	122 (21.32%)	3 (0.52%)	131 (22.9%)	
	Total	23 (4%)	525 (91.78%)	24 (4.19%)	572 (100%)	
Expressing your opinion about the operation to the surgeon	Illiterate	21 (3.6%)	98 (17.13%)	32 (5.59%)	151 (26.3%)	0.189
	Basic education	59 (10.3%)	188 (32.78%)	43 (7.51%)	290 (50.6%)	
	Higher education	18 (3.1%)	90 (15.73%)	23 (4%)	131 (22.9%)	
	Total	98 (17.1%)	376 (65.73%)	98 (17.13%)	572 (100%)	
Plenty of time was not given for discussing before signing	Illiterate	101 (17.6%)	41 (7.16%)	9 (1.5%)	151 (26.3%)	0.001
	Basic education	192 (33.5%)	58 (10.13%)	40 (6.99%)	290 (50.6%)	
	Higher education	105 (18.35%)	18 (3.14%)	8 (1.39%)	131 (22.9%)	
	Total	398 (69.5%)	117 (20.45%)	57 (9.96%)	572 (100%)	
Details of the form explained before signing	Illiterate	55 (9.6%)	96 (16.78%)		151 (26.3%)	0.829
	Basic education	106 (18.5%)	184 (32.16%)		290 (50.6%)	
	Higher education	44 (7.6%)	87 (15.2%)		131 (22.9%)	
	Total	205 (35.8%)	367 (64.16%)		572 (100%)	
The reason for surgery discussed	Illiterate	6 (1%)	145 (25.14%)		151 (26.3%)	0.792
	Basic education	7 (1.22%)	283 (49.47%)		290 (50.6%)	
	Higher education	2 (0.34%)	129 (22.55%)		131 (22.9%)	
	Total	15 (2.62%)	557 (97.37%)		572 (100%)	
The anesthesia risks discussed	Illiterate	96 (16.78%)	55 (9.67%)		151 (26.3%)	< 0.001
	Basic education	123 (21.5%)	167 (29.19%)		290 (50.6%)	
	Higher education	38 (6.64%)	93 (16.25%)		131 (22.9%)	
	Total	257 (49.3%)	215 (37.58%)		572 (100%)	
The type of anesthesia discussed	Illiterate	49 (8.56%)	102 (17.83%)		151 (26.3%)	< 0.001
	Basic education	51 (8.91%)	239 (41.78%)		290 (50.6%)	
	Higher education	18 (3.14%)	113 (19.75%)		131 (22.9%)	
	Total	118 (20.6%)	454 (79.37%)		572 (100%)	
The risks and complications of surgery are discussed	Illiterate	71 (12.4%)	80 (13.98%)		151 (26.3%)	< 0.001
	Basic education	116 (20.2%)	174 (30.41%)		290 (50.6%)	
	Higher education	34 (5.9%)	97 (16.95%)		131 (22.9%)	
	Total	221 (38.6%)	351 (61.36%)		572 (100%)	

There was a significant statistical difference in comparing answers concerning public/private hospitals, illustrated in Table 5.

**Table 5:** Private-public entities regarding consent details

		neutral	no	Yes	Total	*P* value
The patient trusts the surgeon’s decision	private	0	45 (7.87%)	143 (25%)	188 (32.87%)	0.731
	Public	0	97 (16.96%)	287 (50.17%)	384 (67.13)	
	Total	0	142 (24 0.83%)	430 (75.17%)	100 (100%)	
Feeling comfort with the surgeon	private	16 (2.80%)	6 (1.05%)	166 (29.02%)	188 (32.87%)	0.662
	Public	29 (5.07%)	18 (3.15%)	337 (58.92%)	384 (67.13)	
	Total	45 (7.87%)	24 (4.20%)	503 (87.94%)	100 (100%)	
There was not plenty of time for discussion	Private	9 (1.57%)	149 (26.05%)	30 (5.24%)	188 (32.87%)	< 0.001
	Public	48 (8.39%)	249 (43.53%)	87 (15.21%)	384 (67.13)	
	total	57 (9.97)	398 (69.58%)	117 (20.45%)	100 (100%)	
Knowing the operating surgeon	Private		7 (1.22%)	181 (31.64%)	188 (32.87%)	< 0.001
	Public		46 (8.04%)	338 (59.09%)	384 (67.13)	
	total		53 (9.27%)	519 (90.73%)	100 (100%)	
Alternative procedure discussed	Private		89 (15.56%)	99 (17.31%)	188 (32.87%)	0.77
	Public		212 (37.06%)	172 (30.07%)	384 (67.13)	
	total		301 (52.62%)	271 (47.38%)	100 (100%)	
The anesthesia risk discussed	Private		61 (10.66%)	127 (22.20%)	188 (32.87%)	< 0.001
	Public		196 (34.27%)	188 (32.87%)	384 (67.13)	
	total		257 (44.93%)	315 (55.07%)	100 (100%)	
Knowing the anesthetist	Private		120 (20.98%)	68 (11.89%)	188 (32.87%)	< 0.001
	public		310 (54.20%)	74 (12.94%)	384 (67.13)	
	total		430 (75.17%)	142 (24.83%)	100 (100%)	
the cost of treatment discussed	Private		19 (3.32%)	169 (29.55%)	188 (32.87%)	< 0.001
	public		194 (33.92%)	190 (33.22%)	384 (67.13)	
	total		213 (37.24%)	359 (62.76%)	100 (100%)	

## Discussion

The patient is central to surgical management, which is an invasive procedure with potential complications, including mortality. Therefore, the patient should be informed about every aspect of the procedure, from the identity of the operating surgeon to all details of the perioperative period. Most participants asserted that they thoroughly understood this information; compared to the study done, only 38% of their participants were satisfied with the information given during consent [7].

Around 60% of patients in our study confirm that the operating surgeons review the procedure details and obtain their consent. This aligns with the views of participants from Egypt, where about 89% of physicians and 75% of patients believe that acquiring a signed consent form is the responsibility of the physicians’ task [5]; conversely, 27.8% of the consent was obtained by nurses, According to a study that was carried out in Kirkuk, which is one of the cities that are located close to the region, more than three-quarters of surgical consents were collected by nurses [8]. In contrast, research conducted in a tertiary hospital in Pakistan indicated that all consents were secured by physicians, surgeons, or surgical trainers [6]. Although no explicit regulations exist in our region about who is accountable for obtaining consent, it is generally understood that the surgeon doing the operation is the most appropriate to discuss the procedure, as noted in international reviews [9]0.58.6% of respondents indicated that surgeons provide explanations of the surgery before obtaining consent. Approximately 16% reported that junior doctors, rather than surgeons, deliver this information. Furthermore, a study found no statistically significant difference between the explanations provided by surgeons and those given by junior doctors [10].

While a lack of statistical significance was observed in academic education levels concerning the explanation of the consent form, consistent with a study conducted in Muscat in 2024, 35% of participants in our study reported inadequate explanation of the form, compared to 22% of participants in the Muscat study who expressed similar concerns [11], Most research participants in Pakistan assert that inadequate time was allocated for consent, with no statistically significant difference between illiterate and literate individuals. Conversely, our study revealed a significant statistical difference in educational levels (*P* value < 0.001), with most participants indicating insufficient time was permitted [6].

Approximately 60% of our participants said that surgeons obtained consent, while 27.8% reported nurses obtained it. In a survey conducted among physicians in Croatia, 60% of surgeons believed that it is the physician’s responsibility to get consent, whereas 24% thought it is the nurse’s responsibility [12].

Anesthesia is a crucial component of surgical procedures, and the anesthetist is vital to the surgical team; however, it is noteworthy that three-quarters of our participants are unaware of an anesthetist’s involvement with operations, with the significant statistical difference in educational level (*P* value < 0.001) a considerable number of the participants deny taking information about the type of anesthesia and complication of anesthesia (20% and 38%) respectively, comparing to other studies 26% and 83% of participants satisfy about anesthesia details [6, 7].

In our region, the coexistence of public and private hospitals necessitates a comparison of consent-taking practices across these sectors. The cost of surgery is particularly critical in the private sector. Our analysis revealed a statistically significant difference in patients’ awareness of surgical costs, with 37% of respondents unaware of the price. Additionally, there was a similar statistically significant difference regarding the information provided about the surgeon and anesthetist and the opportunity to discuss the procedure. These findings align closely with surveys conducted in other countries comparing both sectors [13, 14].

Before surgery, patients should be informed about the procedure’s expectations, including potential postoperative complications and recovery time. More than half of those surveyed believe this information is discussed during the informed consent process, which is a higher percentage compared to a study conducted in Turkey in 2015 on the same topic [15]. The local Kurdistan regional government publishes and distributes a booklet outlining all anticipated common complications across all surgical wards, which is believed to enhance patient satisfaction regarding their information.

Currently, no health insurance is available in our locality, and public hospitals operate under a semiprivate system requiring patients to pay out-of-pocket in addition to their free services. Therefore, patients need to be informed about the surgery cost before the operation, as research indicates that patients in the USA prefer to discuss costs before treatment decisions are made [16]; approximately 38% of participants report not obtaining pricing information, with nearly half of public hospital patients exhibiting the same trend. This finding warrants serious consideration, and decision-makers should guide this matter.

The comparison between the public and private sectors, coupled with the educational attainment of many participants, represents a notable strength of our study concerning others in this field. A significant number of participants in direct interviews identified another key strength: the authenticity of the situation presented.

The lack of participation from the responsible surgeons is considered a limitation, and comparing outcomes and deficiencies with their involvement would have been more advantageous. The electronic aspects of consent in the context of artificial intelligence require greater emphasis in future research endeavors. Comparing various surgical specialties and distinguishing between emergency and elective procedures requires greater attention to detail.

## Conclusion

Although the existing procedure for acquiring informed consent adheres to established norms, and patients’ rights to information regarding their diagnosis and potential complications are appropriately acknowledged, the current situation requires significant enhancement, compelling surgical and anesthetic departments to implement stringent protocols for physicians to follow, especially regarding knowledge of the patient about the anesthesia and anesthetist.

Good knowledge about surgical consent will lead to accepted communication skills, benefitting the patient and the surgeon and protecting their rights.

The Surgical Association, Ministry of Health, and academic colleges should collaborate to develop a standardized informed consent form encompassing all aspects of the surgery, including its nature, anticipated outcomes, and the individuals responsible for the surgical and anesthetic procedures. This initiative should be integrated into community health education, with explicit guidelines for surgeons to allocate sufficient time and provide comprehensive information to patients prior to surgery. Additionally, hospitals should be mandated to meticulously document all relevant details, particularly the costs associated with each surgical procedure.

In the intelligence era, software experts can develop applications and websites to streamline these details.

## Electronic supplementary material

Below is the link to the electronic supplementary material.

Supplementary Material 1

## Data Availability

Availability of data and materials data will be available for everyone, and the raw data attached under the name (Surgical Consent, Perception of the Patients Who Underwent a Surgical Operation in the Kurdistan Region, Iraq), can connect with the corresponding author, Dawan Jamal Hawezy with Gmail dawan.jamal@koyauniveristy.org to have raw data when requested.
