# Supplementary material for: Surgical consent, perception of the patients who underwent a surgical operation in the Kurdistan region, Iraq
Source: BMC Med Ethics. 2025 May 2;26:55. doi: 10.1186/s12910-025-01218-0 (PMC12046714; doi:10.1186/s12910-025-01218-0)
Supplement: Supplementary file 1 — Supplementary Material 1 [file 12910_2025_1218_MOESM1_ESM.pdf]

Surgical Consent, Perception of the Patients Who Underwent a Surgical Operation in the Kurdistan Region, Iraq

Greetings, students. Please ensure that all questions are spoken in the patient's mother language after consent is obtained. After that, interpret their responses to ensure they are adequate and complete out this form.

* Indicates a required question

Part one

About Patient

Put all the information that you collected.

1. Patient name *

2. Age *

3. Sex *

Mark only one oval.

- Male
- Female

4. Date of Operation *

Example: January 7, 2019

5. Marital State *

Mark only one oval.

- Married
- Single
- divorced

6. Education Level *

Mark only one oval.

- *Illiterate*
- *Basic education from primary school to the university*
- *Higher education*

7. Occupation *

Mark only one

- Academic staff
- Employer
- Housewife
- Retired
- Student
- Worker
- None

8. Type of Surgery *

Mark only one oval.

- ⬭ Open
- ⬭ Laparoscopy

9. Type of anesthesia *

Mark only one oval.

- ⬭ General
- ⬭ Local
- ⬭ Spinal anesthesia

10. Hospital *

Mark only one oval.

- ⬭ Public
- ⬭ Private

11. **Past Medical History** *

Check all that apply

- ○ None
- ○ Diabetes mellitus
- ○ Cardiovascular disease, including **hypertension**
- ○ Respiratory disease
- ○ Neurological disease

12. **Past Surgical History:** Numbers of surgeries done before *

____

13. Time of Interview concerning operation time (how many hours or days after surgery) *

____

14. Who signs as a relative? *
Apart from the patient, as per Iraq law, one of the relatives should sign as a witness during the informed consent; please specify who this relative.

Mark only one oval.

- Him/ Her Parents
- Husband/Wife
- Son/Daughter
- Brother/Sister
- None

15. a healthcare worker who explains the surgical informed consent process *

Mark only one oval. Please define all of these for the patient before choosing.

- *Nurse*
- *Junior house officer*
- *Senior house officer*
- *The surgeon responsible for the surgery*
- *None*

16. Time of taking consent *
When informed consent is taken concerning the time of surgery

Mark only one oval.

- ( ) Days before the operation
- ( ) Hours before the operation
- ( ) Inside the theater
- ( ) Postoperatively

Part two

Regarding the patient-surgeon relationship

We mean by the surgeon, the surgeon who performs the procedure; please explain this to the patient clearly.

17. Patient trusts surgeon's decision regarding the procedure * (how much the patient trusts the surgeon who performs his/her surgical procedure).

Mark only one oval.

- ( ) Never
- ( ) Seldom
- ( ) Sometimes
- ( ) Often
- ( ) Always

18. Feeling comfortable with the Surgeon who performs the procedure

Mark only one oval.

- ( ) Never
- ( ) Seldom
- ( ) Sometimes
- ( ) Often
- ( ) Always

19. Respecting your Surgeon's opinion *

Mark only one oval.

- Never
- Seldom
- Sometimes
- Often
- Always

20. Expressing your concerns about the operation to the surgeon *

Mark only one oval.

- Never
- Seldom
- Sometimes
- Often
- Always

21. Feeling that the surgeon heard and understood your views and concerns *

Mark only one oval.

- Never
- Seldom
- Sometimes
- Often
- Always

22. There was not plenty of time for discussion before signing the form *

Mark only one oval.

- Never
- Seldom
- Sometimes
- Often
- Always

Answer by Yes or No

Please clarify each question for the patient in simple language before getting the answer.

23. Apart from this time, did you sign the informed consent before as a patient or relative? *

Mark only one oval.

- Yes
- No

24. Did the patient read the informed consent before signing? *

Mark only one oval.

- Yes
- No

25. Before the patient signs the informed consent, does the form explain for her/him or not? *

Mark only one oval.

- Yes
- No

26. Does the patient know which surgeon operates? *

Mark only one oval.

- Yes
- No

27. Knowing the reason for surgery *

Mark only one oval.

- Yes
- No

28. Knowing the type/nature of surgery done *

Mark only one oval.

- Yes
- No

29. Alternative procedure discussed give example *

Mark only one oval.

- Yes
- No

30. Conservative management discussed. Explain the meaning of conservative. *

Mark only one oval.

- Yes
- No

31. Knowing the options for alternative treatment *

Mark only one oval.

- ( ) Yes
- ( ) No

32. Knowing the anesthesia risks *

Mark only one oval.

- ( ) Yes
- ( ) No

33. Knowing the type of anesthesia *

Mark only one oval.

- ( ) Yes
- ( ) No

34. Knowing anesthetist *

Mark only one oval.

- ( ) Yes
- ( ) No

35. Knowing the risks and complications of surgery *

Mark only one oval.

- ( ) Yes
- ( ) No

36. Knowing the expected time, the surgery will take *

Mark only one oval.

- Yes
- No

37. Knowing the postoperative care *

Mark only one oval.

- Yes
- No

38. Knowing what to eat after surgery *

Mark only one oval.

- Yes
- No

39. Knowing when to resume working *

Mark only one oval.

- Yes
- No

40. Knowing the cost of treatment *

Mark only one oval.

- Yes
- No

41. Did he accept that surgeons can ask for help from others? * explain before getting the answer

Mark only one oval.

- ( ) Yes
- ( ) No

This content is neither created nor endorsed by Google.

Google Forms
